# Dietary Intervention Is Associated with Lower CGM-Derived Glucose Rate-of-Change in Healthy Young Women: A Pilot Fixed Sequential-Intervention Study

**DOI:** 10.3390/nu18142374

**Published:** 2026-07-21

**Authors:** Agnieszka Lejk, Karolina Myśliwiec, Jędrzej Chrzanowski, Maria Skalska, Anna Stefanowicz-Bielska, Malwina Musiał-Paździor, Marta Herstowska, Zuzanna Margas, Kinga Drzewińska, Ilona Iwaszko, Krzysztof Szewczyk, Barbara Pernak, Julia Kropidłowska, Jędrzej Antosiewicz, Wojciech Fendler

**Affiliations:** 1 Department of Pediatrics, Diabetology and Endocrinology, Medical University of Gdansk, 80-211 Gdansk, Poland; 2 Clinic of Arterial Hypertension and Diabetology, University Clinical Center of Gdansk, 80-952 Gdansk, Poland; 3 Department of Biostatistics and Translational Medicine, Medical University of Lodz, 91-347 Lodz, Poland; 4 Division of Internal and Pediatric Nursing, Institute of Nursing and Midwifery, Faculty of Health Sciences with the Institute of Maritime and Tropical Medicine, Medical University of Gdansk, 80-211 Gdansk, Poland; 5 Department of Pediatrics, Diabetology and Endocrinology, University Clinical Center in Gdansk, 80-952 Gdansk, Poland; 6 Department of Bioenergetics and Physiology of Exercise, Medical University of Gdansk, 80-211 Gdansk, Poland; 7 Faculty of Medicine, Medical University of Gdansk, 80-211 Gdansk, Poland

**Keywords:** continuous glucose monitoring, rate of change, glucose dynamics, dietary intervention, glycemic variability, healthy adults

## Abstract

Continuous glucose monitoring (CGM) interpretation in people without diabetes is expanding beyond general glycemic control toward more dynamic metrics, such as Rate of Change (RoC). In this fixed-sequence pilot study conducted in 30 healthy adult females, we evaluated associations between two structured 14-day lifestyle interventions and free-living conditions using standard CGM-derived glycemic variability metrics and a novel framework based on the rate-of-change (RoC) metrics and data clarity using an unblinded Abbott FreeStyle Libre 2 monitoring. The dietary intervention (calorie-tailored low-glycemic index regular meals) were associated with lower time spent in rising (+1 to +2 mg/dL/min) and falling (−2 to −1 mg/dL/min) RoC bins by 4.76 ± 1.87% (complete-case delta, *p* ≤ 0.0071), whereas the 14-day physical activity intervention showed smaller and less consistent RoC changes (0.41 ± 2.20, *p* ≥ 0.3549). These findings, despite some major study design limitations, suggest that RoC may be more responsive and sensitive to short-term dietary pattern modifications in normoglycemic women.

## 1. Introduction

Continuous glucose monitoring (CGM) has become a gold standard for providing clinical care in patients with diabetes. Recently, it was increasingly used to characterize glucose patterns in people without diabetes and for general health and wellness purposes [1]. CGM can provide clinical insights for metabolic risk assessment, preventive cardiology, sports performance, and personalized nutrition [2]. Individual glycemic responses to the same meal can vary widely, and these differences are only partly predicted by conventional anthropometric or biochemical markers [3]. At the same time, interpretation standards for people without diabetes are still evolving, and the normative ranges for CGM metrics are yet to be connected with clinical outcomes.

Traditional CGM metrics, such as time-in-range (TIR), coefficient of variation (CV), mean amplitude of glycemic excursions (MAGE), and glucose management indicator (GMI), summarize average glycemic control and variability, but may not fully capture the temporal structure of glucose dynamics [4,5]. A physiological reference range for CGM-derived glucose rate of change (RoC) has recently been proposed, with a ±2 mg/dL/min over 15 min suggested as a practical upper bound of normal glycemic dynamics in people without diabetes [6]. Rapid glucose changes, glucodensity, and other distributional or temporal metrics may capture complementary information that conventional variability metrics can miss [4,7]. Whether lifestyle interventions can detectibly shift RoC within the normoglycemic population, and whether those changes have clinical implications, remains to be investigated.

Glucose trajectories may be affected by many different actions. While standard glycemic variability metrics are more indicative of steady-state glucose changes, the RoC metrics are particularly well-suited for assessing acute glycemic response. However, while there is comprehensive evidence on acute glycemic changes in people with diabetes, less data has been recorded from healthy individuals.

The two notable features that dynamically affect glucose levels are the postprandial glucose response and acute physical activity compensation. Meal timing, frequency, and composition (specifically the glycemic index, GI) are well-known determinants of postprandial glucose dynamics. A structured low-GI dietary plan has been demonstrated to slow carbohydrate absorption, flattening postprandial glucose curves and reducing both the meal-related glucose rise as well as the reactive fall that follows [8,9]. Regular meal timing may further help by spreading carbohydrate intake more evenly across the day and limiting large excursions. Physical activity affects glucose regulation through different mechanisms, including increased skeletal muscle glucose uptake, improved peripheral insulin sensitivity, and greater GLUT-4 transporter expression [10].

We therefore conducted this prospective pilot study in healthy young women to determine whether two consecutive structured 14-day lifestyle interventions—high-intensity circuit training (HICT; 30 min sessions three times per week) and a calorie-tailored, low-GI five-meal dietary plan—were associated with changes in CGM-derived RoC compared with a habitual-lifestyle baseline period. The primary objective was to assess intervention-associated changes in RoC distributions. Secondary and exploratory objectives were to compare the responsiveness of RoC with conventional glycemic variability (GV) indices and to determine whether the observed RoC distributions were consistent with the recently proposed normative RoC framework.

## 2. Materials and Methods

This prospective, unblinded, fixed-sequence interventional pilot study was conducted among healthy female student volunteers at the University Clinical Center of Gdansk. All participants completed the standardized study phases in the same order: a habitual-lifestyle baseline period under free-living conditions, followed by a physical activity intervention and then a dietary intervention. Details of the interventions are provided in the Supplementary Materials. No randomization or washout period was included. The Bioethical Commission of the Medical University of Gdansk (No. KB/55/2024) approved the study protocol. The principal investigator informed each participant about the study protocol, and all participants provided written consent.

We enrolled 30 female medical students aged 20–30 years with normal body weight and no chronic diseases. Eligibility was targeted to participants who self-reported suboptimal eating habits and insufficient regular physical activity at baseline. Exclusion criteria included health conditions that could interfere with the intervention or the CGM interpretation, including food allergies or dietary restrictions preventing adherence to the study diet, excess body weight, regular structured physical activity before enrollment, acute or chronic disease, medication use affecting glucose metabolism, pregnancy, or lack of informed consent.

Approximately one week before enrollment, each student underwent biochemical testing to assess their health status. On the morning of the study, body composition was assessed using a Tanita InBody 270 analyzer (Tanita, The Netherlands) after a 12 h fast. A nurse then fitted each participant with an Abbott FreeStyle Libra 2 (Abbott, IL, USA) continuous glucose monitoring system and instructed them on how to change the sensor at home. Additionally, all students received a Polar Vantage accelerometer (Polar Electro, Finland) to monitor their physical activity throughout the study.

During the first two weeks, participants were asked to maintain their habitual eating patterns and avoid initiating regular exercise above their habitual activity. During the next two weeks (physical activity intervention), they maintained their habitual diet but introduced regular physical activity in the programme tailored to their baseline fitness level. An instructional video was recorded to guide them through their physical activity. In the final two weeks of the study (dietary intervention), participants received an individualized dietary meal plan. Caloric intake was tailored for each participant, and the plan consisted of five low-glycemic-index meals to be consumed approximately every three hours. After the study was completed, body composition was re-analyzed. Notably, no adverse events have been reported by the participants within the study period.

### 2.1. Blood Collection

Biochemical tests were performed on each participant, including fasting glucose, fasting insulin, glycated hemoglobin, lipid profile (low and high-density lipoprotein, triglycerides), liver enzymes, and vitamin D.

#### 2.1.1. Body Composition Analysis

We performed a body composition analysis on each participant using the Tanita InBody 270 analyzer (Tanita, The Netherlands) after a 12 h fast. Based on this analysis, we obtained the following parameters: body weight (kg), lean body mass (kg, %), body fat percentage (kg, %), skeletal muscle mass (kg, %), and body water percentage (kg, %). Basal metabolic rate (BMR) was not measured directly; it was estimated from fat-free (lean body) mass using the Cunningham equation, also known as the Katch–McArdle formula [11]. Body composition testing was performed both at the study start and conclusion.

#### 2.1.2. Continuous Glucose Monitoring

We used CGM system (Abbott FreeStyle Libre gen 2, Abbott, IL, USA) to measure glycemic variability metrics: mean sensor glucose, coefficient of variation (CV), time below, in and above ranges (54, 70, 140, 180, 250 mg/dL), glycemia risk index (GRI), low and high blood glucose index (LBGI/HBGI), glucose management indicator (GMI), mean amplitude of glycemic excursions (MAGE), area under the curve (AUC), hypo- and hyper-glycemic episodes.

#### 2.1.3. Monitoring of Physical Activity

Participants’ daily physical activity was assessed using an accelerometer integrated into the Polar Vantage M3 sports watch (Polar Electro, Finland). The device was also used to record training data during each HICT session, including mean heart rate (HR), maximum heart rate (HRmax), and training duration. These data were used to support adherence assessment.

High-intensity circuit training (HICT) was performed according to the recommendations of the American College of Sports Medicine (ACSM) [12] and based on modified protocols in previously published studies [13,14]. The training plan consisted of a circuit of 12 exercises: jumping jacks, squats, oblique abdominal crunches, plank, A-skip, lunges, biceps curl, abdominal crunches, dumbbell overhead press, wall sit, bird-dog exercise, and leg scissors. The approach integrates aerobic and resistance exercises into training sessions lasting approximately 7 min. Each HICT session consisted of three circuits separated by a 2 min rest interval. The total duration of a single training session was approximately 30 min.

Before the main experiment, we prepared an instruction video with music and time visualization. One week before the experiment began, participants were familiarized with the training session design. During the training programme, participants trained individually at home and were asked to complete 6 sessions (3 per week, with at least 1 day between sessions).

All participants were monitored using Abbott FreeStyle Libre gen 2 sensors (Abbott, IL, USA), with 15 min interval sampling. Each CGM sensor was certified to provide 14-day data coverage. To cover the study period, each participant was provided with 3 CGM sensors, trained in sensor insertion and troubleshooting, and instructed to contact the study coordinator in case of any technical difficulties or medical events. Only data from participants with at least 70% CGM sensor wear time was included. Standard glycemic variability (GV) indices [6] were computed using GlyCulator 3.0 [7]. RoC values were calculated as described by Richardson [8] over 15 min intervals and categorized into directional and magnitude-based bins. Due to expected data loss, the primary testing framework was based on a linear mixed model with participants as a random intercept and intervention period as a fixed effect, using all available data from participants meeting the wear-time threshold. Complete-case paired Student’s *t*-test was presented as a sensitivity analysis. Two-sided alpha threshold was set at 0.05, and multiplicity was addressed with Bonferroni-Hochberg false discovery rate (FDR) correction. For complete-case delta values, 95% confidence intervals, and the standardized paired effect sizes were reported. Statistical analysis was performed in Python 3.11 with the statsmodels package (v0.14.4).

## 3. Results

The study comprised 30 women, aged 23.47 ± 2.34 years, with HbA1c 5.15 ± 0.23% and BMI 21.88 ± 2.00 kg/m^2^. Detailed clinical characteristics are provided in Table 1.

**Figure 1:**
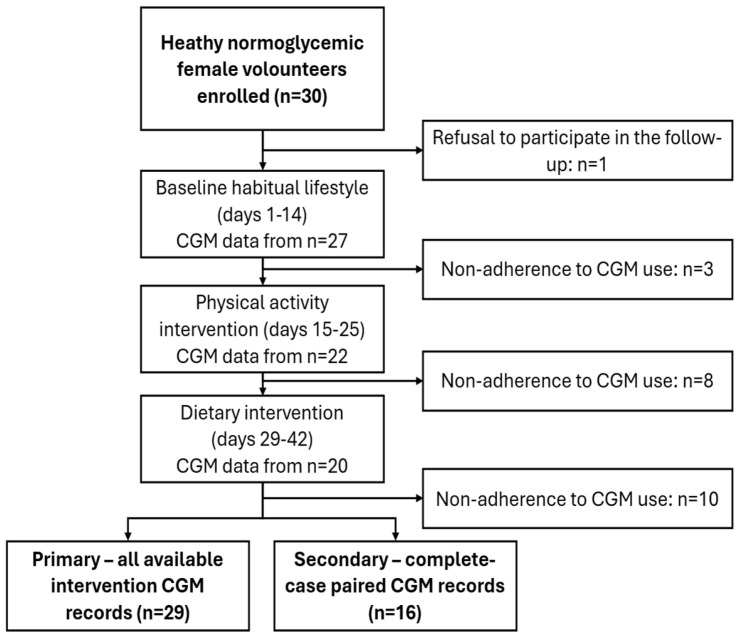
Patient flowchart.

CGM data of sufficient quality were available from 27 patients at baseline, 20 during dietary intervention, and 22 during physical activity intervention; 16 participants had complete data across all three periods (Figure 1).

Pre- to post-intervention changes in body mass and structure are provided in Table 2. Total body weight decreased by a mean of 1.14 ± 1.46 kg over the course of the full study period. Adherence to physical activity was high, with a per participant actigraphy-confirmed median training session count of 6 (IQR: 6–6). median workout session duration of 29 (28–30) minutes and peak HR of 196 (195–197).

For standard GV indices, significant changes with diet were observed in mean sensor glucose, CV, GMI, MAGE, AUC, and the count of hyperglycemic level 1 episodes. Physical activity significantly changed the time above range >180 mg/dL, while the HBGI was affected by both interventions. After FDR correction, only HBGI and MAGE remained significant for the dietary intervention in the mixed-model analysis. While similar differences were observed for complete-case paired *t*-test analysis, none of the classic glycemic variability indices remained significant after FDR correction (Table S2). For details, see Tables S1 and S2.

**Figure 2:**
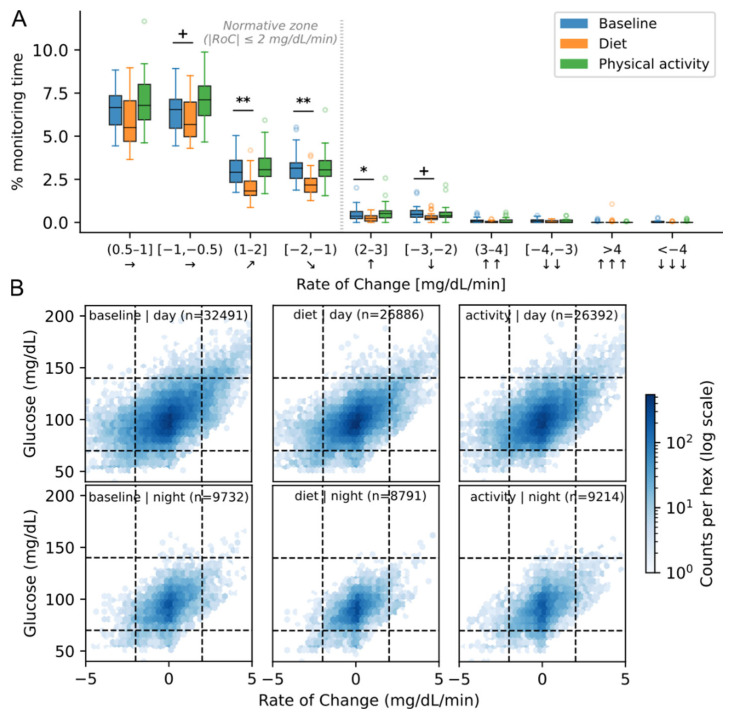
(**A**) Distribution of % monitoring time across glucose rate-of-change (RoC) bins by study period. The dotted vertical line delineates the normative zone (|RoC| ≤ 2 mg/dL/min). (**B**) Phase-space distribution of glucose concentration versus RoC across interventions and time of day (day: 6:00 a.m. to 10:59 p.m.; night: 11:00 p.m. to 5:59 a.m.). ** FDR < 0.01, * FDR < 0.05, + *p* < 0.05. Arrows shows the change in comparison to normative zone of RoC.

In contrast, glucose RoC distributions showed a consistent difference only under dietary intervention, with significance across the normative range (Table 3, Figure 2A). Moreover, the effect remained significant in the complete-case *t*-test analysis and after FDR correction for dietary intervention and RoC change in the +1 to +2 mg/dL/min and −1 to −2 mg/dL/min ranges. This was associated with greater standardized effect size for the +1 to +2 mg/dL/min (complete-case, delta −1.05 percentage points, 95% CI −1.49 to −0.61; standardized effect of −1.27), the −2 to −1 mg/dL/min bin (−0.85% points, 95% CI −1.27 to −0.43; −1.08), and the +2 to +3 mg/dL/min bin (−0.23% points, 95% CI −0.37 to −0.09; −0.88). However, the results must be interpreted with caution; the reported statistical significance does not necessarily translate into a clinically relevant effect, especially for normoglycemic patients.

To assess whether the prandial glucose response was the principal contributor to the RoC changes under dietary intervention, we performed a sensitivity analysis using the daytime and nighttime RoC. We observed no significant differences in nighttime RoC between interventions (all *p* > 0.15), while the daytime RoC confirmed the effect observed for whole-day CGM in dietary, but not the physical, intervention (diet FDR < 0.05 for 1–2 mg/dL/min increase and decrease; PA FDR > 0.5 across all RoC ranges), consistent with the postprandial interpretation (Table S3, Figure 2B). However, because the intervention order was not randomized, these analyses should be interpreted with caution; the effects of interventions might have accumulated over time (adherence drop, metabolic changes, weight loss) or be affected by periodic confounders (phase of menstrual cycle, weather patterns).

## 4. Discussion

We are the first study to investigate changes in CGM-derived glucose RoC in healthy, normoglycemic women during lifestyle interventions. By applying the recently described normative RoC framework [6], we were able to detect intervention differences that would otherwise have been missed. RoC demonstrates an intuitive metric, a proxy for the time in “arrow trends”, which are already available for CGM users [15]. While traditional GV indices showed only modest changes, RoC distributions shifted systematically during dietary intervention and demonstrated large, standardized effect sizes, suggesting that rate-based or distributional metrics can capture non-redundant information relative to standard metrics [7,16] and might be more sensitive under specific scenarios. However, because the study was small and the intervention sequence was fixed, the results should be viewed as hypothesis-generating rather than as causal evidence that dietary intervention produced a beneficial metabolic adaptation [17].

Our study provides novel evidence that a structured dietary intervention, combining calorie tailoring, low-glycemic-index foods, and regular five-meal timing, was associated with a lower CGM-derived glucose RoC in healthy, normoglycemic women. This RoC change, together with significantly lower MAGE and HGBI after FDR correction, and the greater RoC difference during daytime hours, suggests that the RoC changes were most likely related to postprandial glucose. Calorie-tailored, regular, and low-GI foods reduce the rate of gastrointestinal carbohydrate absorption and flatten the glycemic response curve [8,9], directly decreasing the rapidity of prandial glucose rises. The meal schedule might have also independently reduced the carbohydrate bolus per meal, attenuating both the prandial peak and the subsequent reactive fall [18]. The absence of a significant difference in nighttime RoCs supports this intuitive notion [19].

The importance of using a glucose monitoring system in healthy individuals is increasingly being recognized. Recent publications emphasize the current use of parameters such as TTIR (time in tight range), TBR, and TAR. Unfortunately, as discussed in this article, it is emphasized that a short intervention time will not significantly change these parameters [20]. Additionally, there are other factors influencing glucose variability that were not included in this article, such as stress. Numerous publications emphasize its impact on glucose variability observed with CGM [21,22].

On the other hand, the short-term home-based high-intensity circuit training programme showed smaller and less consistent RoC changes, while glycemic variability was more reflected in time above range (180 mg/dL) and HGBI, both of which were lost after FDR correction. This suggests that a physical activity intervention might have a different effect on glucose dynamics, lowering hyperglycemia rather than affecting spikes [23]. This, however, can also be partially attributed to a non-randomized, fixed, sequential design and to either cumulative or periodic confounding of glucose homeostasis.

Prior work demonstrates that the rate of glycemic change influences autonomic and symptomatic responses independently of absolute glucose [24,25]. Thus, even within euglycemia, reducing rapid excursions may be desired. However, we have not evaluated the autonomic or symptomatic responses during interventions, making it difficult to claim the minimal clinically relevant change for RoC metrics. On the other hand, structured physical activity reduced hyperglycemic exposure but did not substantially alter RoC distributions. The apparent lack of early signal should not be over-interpreted as a lack of clinical effect; the physical intervention might have been too short, the participants might not have fully adhered to the training programme, or the total volume might have been insufficient to induce detectable changes in glucose dynamics in healthy young adults [26]. It is possible that a combined dietary and exercise intervention, or a longer training programme allowing full metabolic adaptation, would produce detectable RoC effects.

The dietary effect was most pronounced within the ±2 mg/dL/min normative zone. Despite differences in CGM platforms, sensor generations, and data processing between our dataset and the RoC framework, the normative zone remained relevant, supporting cross-platform generalizability [27]. The sensitivity analysis confirming consistent results across 5 and 15 min resampled data (Table S5) further supports the reliability of sampling interval variation, an important consideration for comparability between CGM devices.

It is crucial to note that the use of CGM in people without diabetes also requires cautious communication. Short-term glucose fluctuations might result in user anxiety or unnecessary dietary restrictions [28]. The device might also cause skin complications such as irritation or allergic contact dermatitis [29]. Finally, only devices that meet the accuracy criteria (eCGM) should be used [20]. Participants and future users should always be instructed to follow manufacturer guidance for sensor placement, rotate sites, avoid application to irritated skin, and seek clinical advice for persistent skin reactions or unexpected glucose patterns. CGM data in healthy individuals should be interpreted as physiological monitoring information rather than as a diagnostic substitute for standard clinical assessment.

The study has several relevant limitations. First, the sample size was small and restricted to young, lean, healthy female students, limiting generalizability to other demographic groups (e.g., males, older adults, overweight individuals, or those with subclinical metabolic dysfunction). Second, all participants completed the same fixed sequence without randomization, counterbalancing, or a washout. Periodic and cumulative effects, adaptation to CGM, other time-related and carryover changes are therefore inseparable across interventions, a major limitation for interpreting our results. The menstrual cycle phase was not recorded, representing a potential source of hormonal confounding given the known influence of estrogen and progesterone on insulin sensitivity and glycemic variability. The CGM missingness was substantial, with only 16 participants having complete data across all periods. Mixed models were used to include the most comprehensive patient cohort; however, we recommend interpreting the results alongside the reported complete-case effect sizes and paired *t*-test significance, accounting for multiple testing corrections. Nonetheless, the large fraction of missing data might indicate potential bias due to adherence, self-selection, or lifestyle behaviours. Dietary adherence was assessed by self-report rather than by weighted food records, dietary recalls, photographs, biomarkers, or other objective assessment methods. The observed dietary intervention changes may reflect combined prescribed intervention, partial adherence, or other concurrent behavioural changes. The exercise phase was home-based, short, and available actigraphy data do not substitute for a fully supervised assessment of achieved intensity. During the intervention, participants’ body weight decreased by 1.14 ± 1.46 kg, which may have partly mediated glucose-dynamic changes. We did not evaluate time-series complexity, entropy, spectral features, or functional/distributional analysis. Finally, established clinical thresholds linking specific RoC distributions to health outcomes in people without diabetes do not yet exist; the physiological and prognostic significance of the observed RoC reductions remains to be determined in randomized and longitudinal studies.

## 5. Limitations

The first limitation of our study was the lack of verification of adherence to the meals provided in the catering diet. We can only trust the study participants and verify this based on reports from the glycemia monitoring system. Secondly, the study was designed so that the exercise programme was followed by the introduction of a nutritional plan. The results of this study should be interpreted with caution. Thirdly, the reduced number of participants accurately represented in the flow diagram may affect the statistical power and precision of the estimated intervention effects. Finally, the superiority of RoC requires confirmation in larger, independent and properly randomized cohorts.

## 6. Conclusions

In healthy young women, a structured dietary intervention (regularly spaced, calorie-tailored, low-glycemic index meals) was associated with lower CGM-derived glucose rate of change in the range of +1 to +2 mg/dL/min and −1 to −2 mg/dL/min, whereas a short-term home-based high-intensity circuit training programme produced less consistent effects. RoC provided complementary information on glucose dynamics not fully captured by traditional glycemic variability indices. These pilot findings suggest potential for the use of RoC as a sensitive metric of metabolic modulation under lifestyle interventions in normoglycemic young adult females.

## Figures and Tables

**Table 1:** Clinical characteristics at baseline (*N* = 30).

	Mean ± SD
Age [years]	23.47 ± 2.34
Sex [female]	100% (30/30)
BMI [kg/m^2^]	21.88 ± 2.00
HbA1C [mmol/mol]	32.76 ± 2.51
HbA1C [%]	5.15 ± 0.23
Glucose [mg/dL]	87.85 ± 5.76
ALT [U/L]	14.08 ± 4.04
Total cholesterol [mg/dL]	171.19 ± 29.15
Triglycerides [mg/dL]	83.59 ± 35.45
HDL cholesterol [mg/dL]	58.52 ± 9.05
LDL cholesterol [mg/dL]	96.04 ± 21.52
Insulin [µIU/mL]	5.72 ± 2.18
25(OH) vitamin D [ng/mL]	28.67 ± 12.85

**Table 2:** The effect of lifestyle interventions on body composition metrics (*N* = 30, mean ± SD).

	Baseline	After the Last Intervention (Diet)	Delta
Weight [kg]	62.09 ± 7.26	60.92 ± 6.85	−1.14 ± 1.46
BMI [kg/m^2^]	21.88 ± 2.00	21.44 ± 1.99	−0.40 ± 0.51
BMR [kcal/day]	1344 ± 101	1334 ± 90	−10.2 ± 23.7
Skeletal Muscle Mass [kg]	24.65 ± 2.68	24.39 ± 2.44	−0.28 ± 0.65
Body Fat Mass [kg]	17.07 ± 4.83	16.30 ± 4.68	−0.69 ± 1.13
Fat Free Mass [kg]	45.03 ± 4.58	44.61 ± 4.18	−0.47 ± 1.10
PBF [%]	27.20 ± 5.41	26.46 ± 5.32	−0.62 ± 1.52
Total Body Water [L]	32.97 ± 3.33	32.66 ± 3.04	−0.36 ± 0.80

**Table 3:** Time spent in Rate of Change [mg/dL/min] over 15 Min intervals (% time spent, mean ± SD). Primary analysis used generalized linear mixed models with a random participant intercept and a fixed intervention slope. Difference from baseline in presented for participants with complete intervention CGM data (*n* = 16). Δ—delta, FDR—false discovery rate, n—count, PA—physical activity. Arrows shows the change in comparison to normative zone of RoC.

	Baseline (*n* = 27)	Diet (*n* = 20)	PA (*n* = 22)	Δ Diet (*n* = 16)	Δ PA (*n* = 16)	Diet *p*	PA *p*
**(0.5, 1.0]: →**	6.64 ± 1.13	5.68 ± 1.39	6.74 ± 1.37	−0.82 ± 1.58	0.37 ± 1.31	0.0551	0.2753
**[−1.0, −0.5): →**	6.59 ± 1.06	5.52 ± 1.01	6.91 ± 1.25	−0.76 ± 1.1	0.8 ± 1.26	0.0141	0.0225
**(1.0, 2.0]:** **↗**	3.05 ± 0.89	1.94 ± 0.72	3.29 ± 0.81	−1.05 ± 0.83	0.24 ± 1.07	0.0001	0.3899
**[−2.0, −1.0):** **↘**	3.23 ± 0.99	2.22 ± 0.71	3.17 ± 0.74	−0.85 ± 0.79	0.08 ± 1.0	0.0001	0.9207
**(2.0, 3.0]: ↑**	0.49 ± 0.4	0.25 ± 0.18	0.56 ± 0.35	−0.23 ± 0.26	0.11 ± 0.31	0.0041	0.1420
**[−3.0, −2.0): ↓**	0.55 ± 0.42	0.33 ± 0.28	0.52 ± 0.37	−0.2 ± 0.34	−0.02 ± 0.24	0.0071	0.8867
**(3.0, 4.0]: ↑↑**	0.11 ± 0.16	0.03 ± 0.05	0.1 ± 0.14	−0.07 ± 0.14	0.02 ± 0.16	0.0569	0.6397
**[−4.0, −3.0): ↓↓**	0.11 ± 0.12	0.07 ± 0.07	0.08 ± 0.1	−0.04 ± 0.12	−0.02 ± 0.10	0.2376	0.3524
**>4.0: ↑↑↑**	0.02 ± 0.04	0.02 ± 0.04	0.01 ± 0.02	0.01 ± 0.06	−0.0 ± 0.03	0.3990	0.6736
**<−4.0: ↓↓↓**	0.04 ± 0.07	0.01 ± 0.03	0.02 ± 0.06	−0.04 ± 0.09	−0.01 ± 0.10	0.1402	0.6954

## Data Availability

The data presented in this study are available on request from the corresponding author due to the subjects’ privacy.

## Supplementary Materials

The following supporting information can be downloaded at: https://www.mdpi.com/article/10.3390/nu18142374/s1, Table S1: Glycemic variability indices across interventions – generalized linear mixed models (GLMM, mean ± SD); Table S2: Glycemic variability indices across interventions–paired Student’s *t*-test (mean ± SD); Table S3: Day- and night-time analysis of CGM RoC (GLMM, mean ± SD); Table S4: Comparison of selected clinical and CGM characteristics depending on data completeness; Table S5: Summary of CGM RoC on 5 and 15-minute resampled data (GLMM, mean ± SD).
