# Supplementary material for: Dietary Intervention Is Associated with Lower CGM-Derived Glucose Rate-of-Change in Healthy Young Women: A Pilot Fixed Sequential-Intervention Study
Source: Nutrients. 2026 Jul 21;18(14):2374. doi: 10.3390/nu18142374 (PMC13414802; doi:10.3390/nu18142374)
Supplement: Supplementary file 1 [file nutrients-18-02374-s001.zip › nutrients-4370776-supplementary.pdf]

Supplementary Materials

Table S1. Glycemic variability indices across interventions—generalized linear mixed models (GLMM, mean±SD).

	Baseline (n=27)	Diet (n=20)	PA (n=22)	Δ Diet (n=16)	Δ PA (n=16)	Diet p	PA p	Diet FDR	PA FDR
**Standard glycemic variability indices**									
**Mean [mg/dL]**	100.74±7.47	96.85±7.65	98.38±7.19	-2.54±3.4	-2.44±5.82	0.0261	0.0851	0.1203	0.2554
**CV [%]**	18.11±3.59	17.1±3.44	17.76±3.54	-1.63±3.68	-0.86±2.72	0.0337	0.3958	0.1412	0.6300
**TBR <54 mg/dL**	0.44±0.77	0.47±0.65	0.5±0.89	0.06±0.73	-0.05±1.18	0.7350	0.8625	0.8991	0.9409
**TBR <70 mg/dL**	3.7±4.98	4.78±9.5	3.47±3.8	1.2±5.87	-0.14±3.61	0.5036	0.8541	0.7633	0.9409
**TIR 70-180 mg/dL**	95.41±5.53	94.44±10.01	95.82±4.17	-1.09±6.19	0.49±4.25	0.6094	0.7460	0.8918	0.8991
**TITR 70-140 mg/dL**	92.04±5.67	92.34±9.76	93.42±3.84	-0.05±7.11	1.58±3.62	0.7585	0.3310	0.8991	0.6207
**TAR >180 mg/dL**	0.44±0.69	0.3±0.44	0.2±0.33	-0.15±0.72	-0.3±0.43	0.2248	0.0192	0.4818	0.1150
**TAR >250 mg/dL**	0.02±0.07	0.0±0.0	0.02±0.1	-0.02±0.09	0.01±0.03	0.2673	0.7501	0.5328	0.8991
**GRI [%]**	10.56±13.76	12.44±21.41	10.01±10.46	2.05±12.06	-0.73±10.94	0.6984	0.8328	0.8991	0.9409
**LBGI**	1.65±1.14	2.0±1.64	1.83±1.16	0.22±0.88	0.18±0.98	0.3651	0.5089	0.6259	0.7633
**HBGI**	0.23±0.21	0.15±0.13	0.17±0.14	-0.08±0.15	-0.07±0.13	0.0039	0.0180	0.0496	0.1150
**GMI [%]**	5.72±0.18	5.63±0.18	5.66±0.17	-0.06±0.08	-0.06±0.14	0.0261	0.0851	0.1203	0.2554
**MAGE**	35.8±9.05	31.87±7.45	34.87±7.25	-5.2±5.94	-2.2±6.06	0.0001	0.1833	0.0020	0.4230
**AUC**	100.28±7.48	95.99±7.61	97.97±7.04	-2.67±3.37	-2.0±5.36	0.0164	0.1285	0.1150	0.3549
**Hypoglycemic extended episodes**	1.22±2.69	1.85±4.64	1.23±2.18	0.94±2.62	0.12±2.33	0.3564	0.9566	0.6259	0.9896
**Hypoglycemic level 1 episodes**	12.11±13.07	14.05±13.84	14.77±13.63	2.62±8.65	4.44±10.83	0.6776	0.2044	0.8991	0.4543
**Hypoglycemic level 2 episodes**	2.07±3.65	2.85±4.45	1.68±2.61	1.5±4.0	-0.25±3.49	0.1544	0.7786	0.3706	0.8991
**Hyperglycemic level 1 episodes**	1.74±3.16	0.95±1.47	1.09±1.8	-0.81±1.76	-0.62±1.09	0.0353	0.0585	0.1412	0.1951
**Hyperglycemic level 2 episodes**	0.07±0.27	0.0±0.0	0.05±0.21	-0.06±0.25	0.0±0.0	0.1376	0.7792	0.3549	0.8991

Table S2. Glycemic variability indices across interventions—paired Student's t-test (mean±SD).

	Baseline (n=16)	Diet (n=16)	PA (n=16)	Δ Diet (n=16)	Δ PA (n=16)	Diet p	PA p	Diet FDR	PA FDR
**Standard glycemic variability indices**									
**Mean [mg/dL]**	99.77±7.53	97.23±8.43	97.33±7.77	-2.54±3.4	-2.44±5.82	0.0092	0.1137	0.0789	0.3249
**CV [%]**	18.77±3.24	17.14±3.79	17.91±3.73	-1.63±3.68	-0.86±2.72	0.0967	0.2243	0.3174	0.4641
**TBR <54 mg/dL**	0.5±0.84	0.57±0.69	0.45±0.79	0.06±0.73	-0.05±1.18	0.7350	0.8625	0.8818	0.9239
**TBR <70 mg/dL**	4.04±5.83	5.24±10.61	3.9±4.22	1.2±5.87	-0.14±3.61	0.4251	0.8777	0.6221	0.9239
**TIR 70-180 mg/dL**	94.94±6.32	93.85±11.15	95.43±4.5	-1.09±6.19	0.49±4.25	0.4915	0.6505	0.6858	0.8485
**TITR 70-140 mg/dL**	91.65±5.77	91.61±10.84	93.24±4.13	-0.05±7.11	1.58±3.62	0.9785	0.1005	1.0000	0.3174
**TAR >180 mg/dL**	0.49±0.74	0.34±0.47	0.19±0.36	-0.15±0.72	-0.3±0.43	0.4130	0.0126	0.6195	0.0940
**TAR >250 mg/dL**	0.02±0.09	0.0±0.0	0.03±0.12	-0.02±0.09	0.01±0.03	0.3332	0.3332	0.5633	0.5633
**GRI [%]**	11.64±15.81	13.69±23.83	10.91±11.19	2.05±12.06	-0.73±10.94	0.5068	0.7940	0.6911	0.8822
**LBGI**	1.79±1.22	2.01±1.84	1.97±1.24	0.22±0.88	0.18±0.98	0.3380	0.4824	0.5633	0.6858

**HBGI**	0.23±0.2	0.16±0.14	0.16±0.15	-0.08±0.15	-0.07±0.13	0.0559	0.0345	0.2134	0.1680
**GMI [%]**	5.7±0.18	5.64±0.2	5.64±0.19	-0.06±0.08	-0.06±0.14	0.0092	0.1137	0.0789	0.3249
**MAGE**	37.21±9.14	32.01±7.87	35.01±8.06	-5.2±5.94	-2.2±6.06	0.0032	0.1676	0.0540	0.3703
**AUC**	98.98±7.24	96.32±8.41	96.98±7.64	-2.67±3.37	-2.0±5.36	0.0064	0.1562	0.0768	0.3703
**Hypoglycemic extended episodes**	1.31±3.14	2.25±5.13	1.44±2.37	0.94±2.62	0.12±2.33	0.1728	0.8333	0.3703	0.9091
**Hypoglycemic level 1 episodes**	11.81±12.38	14.44±15.23	16.25±14.97	2.62±8.65	4.44±10.83	0.2434	0.1219	0.4711	0.3325
**Hypoglycemic level 2 episodes**	1.94±2.72	3.44±4.8	1.69±2.27	1.5±4.0	-0.25±3.49	0.1544	0.7786	0.3703	0.8821
**Hyperglycemic level 1 episodes**	1.81±2.9	1.0±1.51	1.19±2.07	-0.81±1.76	-0.62±1.09	0.0846	0.0364	0.2986	0.1680
**Hyperglycemic level 2 episodes**	0.06±0.25	0.0±0.0	0.06±0.25	-0.06±0.25	0.0±0.0	0.3332	0.7792	0.5633	0.8821
**Time spent in Rate of Change [mg/dL/min] over 15-minute intervals [%]**									
**(0.5, 1.0]: →**	6.39±1.26	5.57±1.47	6.76±1.34	-0.82±1.58	0.37±1.31	0.0551	0.2753	0.2134	0.5162
**[-1.0, -0.5): →**	6.17±1.03	5.41±0.94	6.97±1.33	-0.76±1.1	0.8±1.26	0.0141	0.0225	0.0940	0.1350
**(1.0, 2.0]: ↗**	3.0±0.87	1.95±0.8	3.24±0.9	-1.05±0.83	0.24±1.07	0.0001	0.3899	0.0060	0.6138
**[-2.0, -1.0): ↘**	3.09±0.85	2.24±0.79	3.17±0.75	-0.85±0.79	0.08±1.0	0.0007	0.7495	0.0210	0.8818
**(2.0, 3.0]: ↑**	0.46±0.28	0.24±0.2	0.57±0.36	-0.23±0.26	0.11±0.31	0.0036	0.1682	0.0540	0.3703
**[-3.0, -2.0): ↓**	0.53±0.42	0.33±0.31	0.51±0.41	-0.2±0.34	-0.02±0.24	0.0321	0.7438	0.1680	0.8818
**(3.0, 4.0]: ↑↑**	0.1±0.15	0.03±0.06	0.12±0.16	-0.07±0.14	0.02±0.16	0.0569	0.6397	0.2134	0.8485
**[-4.0, -3.0): ↓↓**	0.11±0.11	0.07±0.08	0.09±0.11	-0.04±0.12	-0.02±0.1	0.2376	0.3524	0.4711	0.5715
**>4.0: ↑↑↑**	0.01±0.04	0.03±0.05	0.01±0.03	0.01±0.06	-0.0±0.03	0.3990	0.6736	0.6138	0.8599
**<-4.0: ↓↓↓**	0.04±0.09	0.01±0.02	0.03±0.07	-0.04±0.09	-0.01±0.1	0.1402	0.6954	0.3657	0.8693

Table S3. Day- and night-time analysis of CGM RoC (GLMM, mean±SD).

	Baseline (n=16)	Diet (n=16)	PA (n=16)	Δ Diet (n=16)	Δ PA (n=16)	Diet p	PA p	Diet FDR	PA FDR
**Time spent in Rate of Change [mg/dL/min] over 15-minute intervals [%] – daytime (6:00 am – 10:59 pm)**									
**(0.5, 1.0]: →**	6.34±1.23	5.47±1.13	6.34±1.1	-0.87±1.27	0.0±0.79	0.0167	0.9922	0.0620	0.9922
**[-1.0, -0.5): →**	6.75±0.82	5.59±0.36	7.98±1.54	-1.15±0.9	1.23±1.0	0.0082	0.0104	0.0520	0.0520
**(1.0, 2.0]: ↗**	3.54±1.06	2.29±1.22	4.07±1.35	-1.26±0.78	0.53±1.08	0.0006	0.1523	0.0064	0.3356
**[-2.0, -1.0): ↘**	3.75±0.98	2.9±1.19	3.84±0.81	-0.85±0.55	0.1±0.49	<0.0001	0.6511	0.0014	0.8139
**(2.0, 3.0]: ↑**	0.63±0.38	0.3±0.31	0.82±0.47	-0.33±0.3	0.19±0.4	0.0186	0.2143	0.0620	0.3896
**[-3.0, -2.0): ↓**	0.72±0.74	0.42±0.49	0.67±0.69	-0.3±0.44	-0.05±0.33	0.0423	0.7196	0.1209	0.8466
**(3.0, 4.0]: ↑↑**	0.18±0.23	0.07±0.1	0.11±0.2	-0.11±0.22	-0.07±0.12	0.1183	0.2758	0.2958	0.4244
**[-4.0, -3.0): ↓↓**	0.13±0.15	0.11±0.1	0.15±0.18	-0.01±0.13	0.03±0.12	0.7844	0.6104	0.8715	0.8139
**>4.0: ↑↑↑**	0.02±0.07	0.05±0.07	0.03±0.05	0.02±0.11	0.0±0.04	0.5697	0.8887	0.8139	0.9355
**<-4.0: ↓↓↓**	0.0±0.0	0.0±0.0	0.0±0.0	-0.0±0.0	-0.0±0.0	0.2413	0.1678	0.4022	0.3356
**Time spent in Rate of Change [mg/dL/min] over 15-minute intervals [%] – nighttime (11:00 pm – 5:59 am)**									
**(0.5, 1.0]: →**	5.05±1.64	3.86±1.76	5.8±1.77	-1.2±2.57	0.75±1.93	0.2295	0.3073	1.0000	1.0000
**[-1.0, -0.5): →**	3.27±1.04	2.87±1.32	3.63±1.68	-0.39±1.75	0.36±1.42	0.5463	0.4919	1.0000	1.0000
**(1.0, 2.0]: ↗**	1.49±1.46	0.8±0.74	1.41±0.92	-0.7±1.41	-0.09±1.76	0.1519	0.8581	1.0000	1.0000
**[-2.0, -1.0): ↘**	0.89±0.25	0.74±0.63	0.87±0.65	-0.16±0.77	-0.03±0.56	0.5822	0.8991	1.0000	1.0000
**(2.0, 3.0]: ↑**	0.14±0.15	0.17±0.26	0.16±0.32	0.03±0.2	0.03±0.26	0.6761	0.7682	1.0000	1.0000
**[-3.0, -2.0): ↓**	0.14±0.15	0.17±0.19	0.17±0.29	0.03±0.25	0.03±0.28	0.7419	0.7520	1.0000	1.0000

**(3.0, 4.0]: ↑↑**	0.03±0.09	0.0±0.0	0.0±0.0	- 0.03±0.09	-0.03±0.09	0.2206	0.2206	1.0000	1.0000
**[-4.0, -3.0): ↓↓**	0.0±0.0	0.0±0.0	0.0±0.0	0.0±0.0	0.0±0.0	1.0000	1.0000	1.0000	1.0000
**>4.0: ↑↑↑**	0.0±0.0	0.0±0.0	0.0±0.0	0.0±0.0	0.0±0.0	1.0000	1.0000	1.0000	1.0000
**<-4.0: ↓↓↓**	0.0±0.0	0.0±0.0	0.0±0.0	0.0±0.0	0.0±0.0	1.0000	1.0000	1.0000	1.0000

Table S4. Comparison of selected clinical and CGM characteristics depending on data completeness

	**Complete-case (n=16)**	**Incomplete (n = 14)**	**p (MWU)**
Age (y)	23.5±1.5	23.4±3.1 (n=14)	0.9384
BMI (kg/m2)	21.2±1.9	22.7±1.9 (n=14)	0.0374
Weight (kg)	61.1±7.6	63.2±6.9 (n=14)	0.4477
Body fat (%)	27.0±6.1	27.4±4.7 (n=14)	0.8173
Fat-free mass (kg)	44.5±5.5	45.6±3.4 (n=14)	0.4932
HbA1c (%)	5.2±0.2	5.1±0.2 (n=11)	0.5256
Fasting glucose	87.8±6.1	87.9±5.5 (n=11)	0.9662
Insulin	5.5±1.4	6.1±3.1 (n=10)	0.5866
HOMA-IR	1.2±0.3	1.3±0.7 (n=10)	0.6159
Triglycerides	67.4±17.4	107.1±42.3 (n=11)	0.012
HDL	59.4±10.0	57.3±7.8 (n=11)	0.5447
LDL	92.1±21.9	101.8±20.6 (n=11)	0.2504
Baseline CGM mean	99.8±7.5	102.1±7.5 (n=11)	0.4294
Baseline CGM CV (%)	18.8±3.2	17.1±4.0 (n=11)	0.2767

Table S5. Summary of CGM RoC on 5 and 15-minute resampled data (GLMM, mean±SD).

**Time spent in Rate of Change [mg/dL/min] over 15-minute intervals [%] – resampled 5 minutes**									
**(0.5, 1.0]: →**	8.49±1.04	6.96±1.29	8.05±1.28	-1.67±1.78	-0.56±1.39	0.0013	0.1154	0.0037	0.1649
**[-1.0, -0.5): →**	8.41±1.21	6.68±0.89	8.09±1.19	-1.6±1.33	-0.13±1.62	0.0001	0.7381	0.0005	0.8201
**(1.0, 2.0]: ↗**	3.7±1.35	2.45±1.06	3.6±0.94	-1.25±1.02	-0.14±1.11	0.0000	0.4757	<0.0001	0.5596
**[-2.0, -1.0): ↘**	3.91±1.29	2.67±1.07	3.58±1.08	-1.17±0.95	-0.36±1.02	0.0000	0.0687	<0.0001	0.1280
**(2.0, 3.0]: ↑**	0.59±0.48	0.24±0.19	0.49±0.42	-0.37±0.41	-0.1±0.28	0.0000	0.2074	0.0003	0.2765
**[-3.0, -2.0): ↓**	0.63±0.5	0.38±0.33	0.46±0.38	-0.26±0.43	-0.18±0.24	0.0006	0.0145	0.0020	0.0363
**(3.0, 4.0]: ↑↑**	0.11±0.12	0.03±0.05	0.06±0.08	-0.06±0.12	-0.03±0.07	0.0704	0.0900	0.1280	0.1500
**[-4.0, -3.0): ↓↓**	0.1±0.12	0.04±0.05	0.08±0.09	-0.08±0.11	-0.05±0.07	0.0003	0.0580	0.0011	0.1280
**>4.0: ↑↑↑**	0.02±0.03	0.01±0.02	0.02±0.04	-0.01±0.03	-0.0±0.06	0.0989	0.8507	0.1522	0.8507
**<-4.0: ↓↓↓**	0.02±0.03	0.01±0.03	0.02±0.07	-0.01±0.04	0.01±0.09	0.2364	0.7983	0.2955	0.8403
**Time spent in Rate of Change [mg/dL/min] over 15-minute intervals [%] – resampled 15 minutes**									
**(0.5, 1.0]: →**	8.37±1.03	7.0±1.29	8.0±1.54	-1.69±1.7	-0.6±1.64	0.0009	0.1508	0.0036	0.2513
**[-1.0, -0.5): →**	8.53±1.16	6.77±1.04	8.11±1.2	-1.53±1.36	-0.13±1.49	0.0000	0.1760	<0.0001	0.2647
**(1.0, 2.0]: ↗**	3.71±1.43	2.38±1.1	3.65±0.94	-1.29±1.07	-0.12±1.14	0.0000	0.6809	<0.0001	0.8010
**[-2.0, -1.0): ↘**	3.87±1.34	2.7±1.1	3.67±1.1	-1.09±1.06	-0.27±1.16	0.0000	0.3015	<0.0001	0.4020
**(2.0, 3.0]: ↑**	0.61±0.49	0.27±0.21	0.48±0.42	-0.37±0.42	-0.11±0.24	0.0001	0.1173	0.0006	0.2346
**[-3.0, -2.0): ↓**	0.62±0.48	0.37±0.35	0.5±0.44	-0.26±0.45	-0.12±0.27	0.0016	0.0938	0.0046	0.2164
**(3.0, 4.0]: ↑↑**	0.1±0.14	0.03±0.06	0.06±0.1	-0.06±0.15	-0.03±0.08	0.0974	0.1853	0.2164	0.2647
**[-4.0, -3.0): ↓↓**	0.09±0.13	0.03±0.05	0.07±0.09	-0.09±0.13	-0.05±0.1	0.0011	0.1411	0.0036	0.2513
**>4.0: ↑↑↑**	0.01±0.03	0.01±0.02	0.01±0.03	-0.0±0.03	-0.0±0.05	0.5566	0.7504	0.6958	0.8338
**<-4.0: ↓↓↓**	0.01±0.03	0.01±0.03	0.02±0.07	0.0±0.03	0.01±0.09	0.9492	0.8098	0.9492	0.8525
